# Circular over-the-wire pulsed field ablation for atrial fibrillation: differences in outcomes between conscious sedation or general anesthesia

**DOI:** 10.1007/s10840-025-02134-4

**Published:** 2025-10-16

**Authors:** M. Van der Graaf, B. G. S. Abeln, V. F. Van Dijk, M. Liebregts, M. C. E. F. Wijffels, J. C. Balt, L. V. A. Boersma

**Affiliations:** 1 Department of Cardiology, St. Antonius Hospital, Koekoekslaan 1, Nieuwegein, 3435 CM The Netherlands; 2 Department of Cardiology, Amsterdam UMC, Amsterdam, The Netherlands

**Keywords:** Pulsed field ablation, Atrial fibrillation, Pulmonary vein isolation, Conscious sedation, General anesthesia, Phrenic nerve stimulation

## Abstract

**Background:**

Pulmonary vein isolation (PVI) using pulsed field ablation (PFA) is typically performed under general anesthesia (GA) due to patient discomfort and pain. However, procedures under GA require specialized personnel for airway management. PFA systems with suitable characteristics may enable PFA-procedures to be performed under bolus- administered conscious sedation (CS).

**Objective:**

Assess the feasibility, safety, and efficacy of performing PVI with a circular-over-the-wire PFA catheter under CS, compared to GA.

**Methods:**

We conducted a single-center registry study of atrial fibrillation (AF) patients undergoing an ablation with the circular over-the-wire PFA catheter, between January 29 and December 31, 2024, at the St. Antonius Hospital, The Netherlands. CS was used if no anesthesiology team was available. Endpoints included acute isolation of ablation targets, procedural characteristics and freedom from adverse events.

**Results:**

The analysis included 174 consecutive patients (67.2% male, mean age 62.8 ± 9.3 years), with 62.1% having paroxysmal AF. GA was used in 132 patients (75.9%). There were no differences in baseline characteristics between the groups. Total number of applications was higher in the GA group: 33.0 [IQR 32.0; 36.0] vs. 32.0 [IQR 32.0; 33.0], *p* < 0.001. Median skin-to-skin procedural time was comparable between groups: GA 39.0 min [IQR 34.0;46.0] vs. CS 42.0 min [IQR 37.5; 46.0], *P =* N.S. Acute procedural efficacy was 100% in both groups. One patient in the GA group experienced a major bleeding complication.

**Conclusion:**

Conscious sedation offers an efficient and safe alternative to general anesthesia for patients undergoing a procedure with use of the circular over-the-wire PFA catheter.

**Graphical Abstract:**

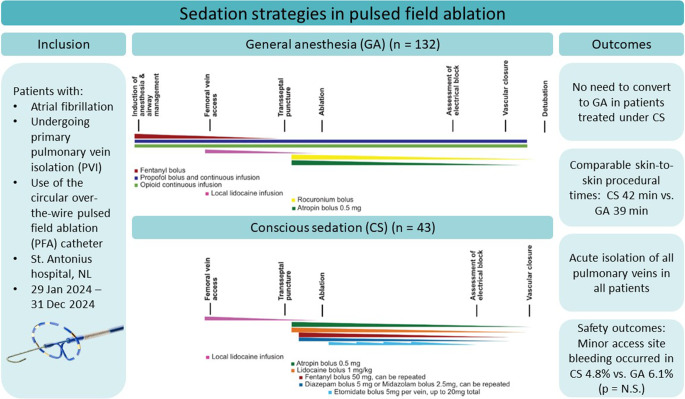

## Introduction

Pulsed field ablation (PFA) has quickly become a widely used therapy for pulmonary vein isolation (PVI) in patients with atrial fibrillation (AF) and has shown to decrease procedure times and avoid well known complications of thermal ablation methods, without compromising efficacy outcomes [1–3]. During PFA procedures, multiple short (milli- to nanosecond) high voltage electrical pulses are delivered to target the myocardium, inducing cell death and fibrosis formation [4]. While complications to surrounding tissues are rare, capture of extra-cardiac structures is a well-known phenomenon during PFA [5]. Capture of these structures, such as the phrenic nerve, diaphragm and skeletal muscles can cause diaphragmatic contractions and coughing [6]. This may lead to patient discomfort and patient movement, potentially necessitating the cessation of the ablation procedure. Consequently, most PFA procedures are performed under general anesthesia (GA) or continuous deep sedation, supervised by a specialized anesthesiology team, to ensure continuous airway management and hemodynamic monitoring. However, despite continuous monitoring, GA and deep sedation carry inherent risks [7]. The requirement for a specialized anesthesiology team results in increased health care utilization and could potentially hamper the adoption of PFA in everyday clinical practice, depending on local legislation and varying availability of anesthetic support in different geographies.

In recent years, several PFA systems have become commercially available, offering different pulse profiles and delivery systems. This variation includes the number of pulses during one train, and pulse characteristics such as voltage, waveform, polarity, and pulse duration. Also, different catheter shapes and number of electrodes may have an influence on energy delivery to the heart and beyond. These settings could influence the degree of phrenic nerve stimulation and discomfort for patients. A recent study with the pentaspline ablation catheter (FARAPULSE, Boston Scientific, Marlborough, USA) demonstrated that PFA under conscious sedation (CS) is feasible [8]. However, high doses of sedatives were used in this study (mean fentanyl dose 209 ± 40 mcg) to ensure patient tolerance during the procedure. Another PFA system, which uses a circular, over-the-wire catheter (PulseSelect, Medtronic, Minneapolis, USA), has a different pulse profile which could potentially make this PFA-method more suitable for procedures under CS.

This study aims to provide an insight into the feasibility of CS-based PFA procedures using the circular over-the-wire catheter, describing a workflow that can be applied in clinical practice and comparing its safety and efficacy to procedures that were performed under GA.

## Methods

### Study design

This is a retrospective analysis of a single-center, observational registry study aiming to investigate the outcomes of performing PFA procedures in everyday clinical practice. The current analysis focused on the safety and efficacy of PFA procedures performed using the PFA system with a circular, over-the-wire catheter without general anesthesia (GA) or continuous deep sedation, compared to those treated with GA. The study was performed in accordance with the Declaration of Helsinki and the observational registry was approved by the local ethics committee and institutional review board.

### Patient population

Patients undergoing a de novo PVI catheter ablation to treat paroxysmal or persistent AF with the circular, over-the-wire catheter PFA system between January 29 and December 31, 2024, in the St. Antonius Hospital, the Netherlands, were included in this study. Patients with a prior left atrial ablation procedure were excluded from this study. The planning department was responsible for patient planning without any influence of physicians, meaning GA was used if the anesthesiology team was available, and CS was used if not. Patients with known severe obstructive sleep apnea or severe pulmonary disease were excluded from conscious sedation procedures. Anticoagulant medication was continued during the procedure in both GA and CS cases.

### Sedation strategy

GA was provided by a specialized anesthesiology team. Induction of GA was typically achieved with the administration of a combination of continuous propofol and opioid infusion intravenously and a dose of a muscle relaxant (rocuronium), in the presence of an anesthesiologist. Airway management was performed using a laryngeal mask in most patients, and endotracheal intubation in a small amount of patients. During the procedure, continuous propofol infusion was used and the patient was monitored by the anesthesiologist and/or a specialized anesthesiology nurse. After the procedure, patients were transferred to the postoperative observation room for additional monitoring.

**Fig. 1:**
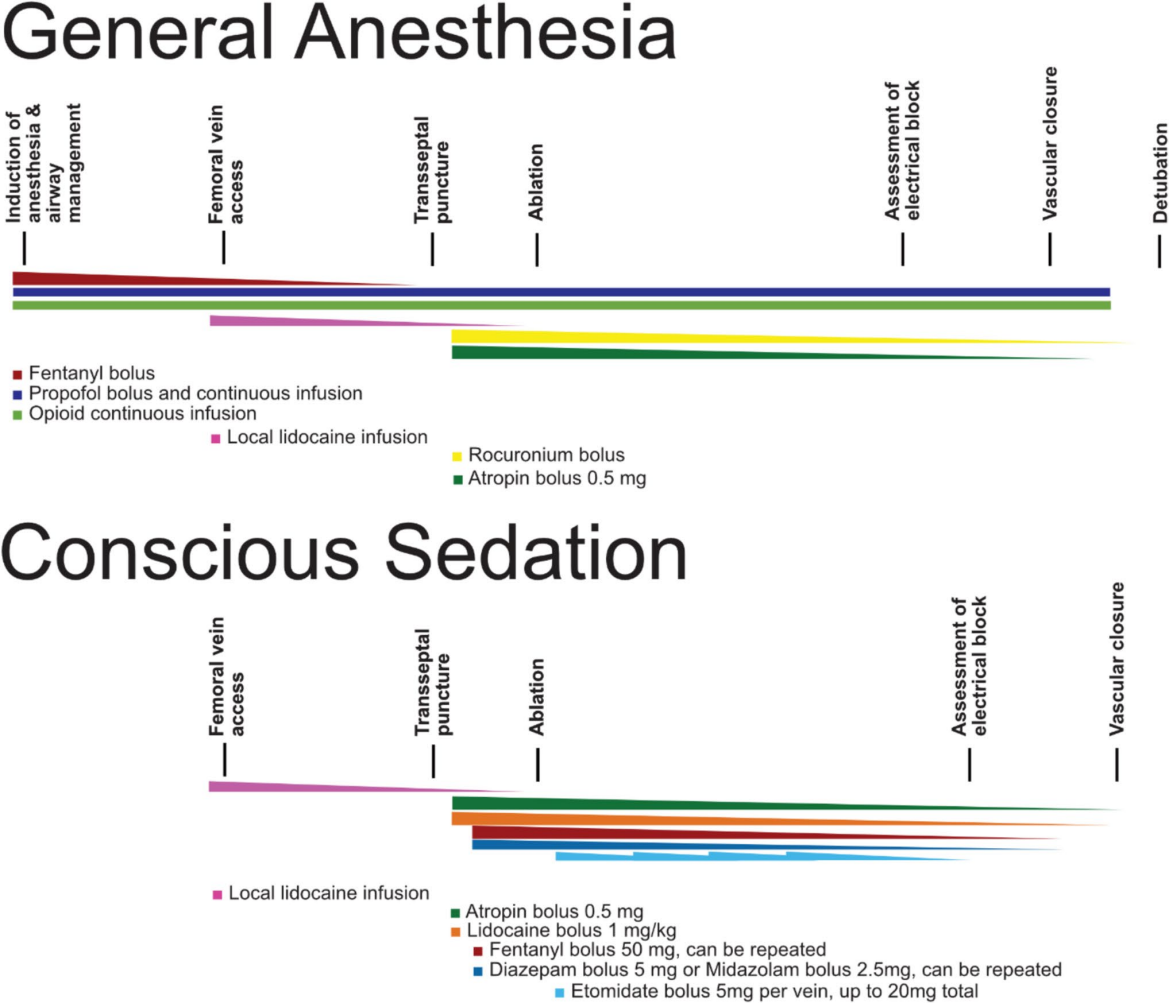
Timeline and schematic overview of medication administration for general anesthesia and conscious sedation

For procedures performed under CS, local anesthetics were delivered at the groin prior to gaining vascular access. Prior to the transseptal puncture, intravenous sedatives were administered by the operator through the side port of the sheath. The sedative medication consisted of a bolus of 50 mg fentanyl and 5 mg diazepam or 2.5 mg midazolam, which could be repeated if the operator deemed the patient was not adequately sedated. Hemodynamic and respiratory monitoring was performed by the personnel of the electrophysiology (EP) laboratory staff, monitoring the pulse oximetry, heart rate, blood pressure, and respiratory rate with a monitoring system (MindRay VS 9, Mindray, China). All staff members were trained in airway management. Prior to the PFA energy applications, the operator administered 0.5 mg of atropine, along with an intravenous bolus of lidocaine of 1 mg/kg to suppress a coughing response [9, 10]. In case of severe and persistent coughing, pain, or patient discomfort due to phrenic nerve capture, a bolus of etomidate of up to 5 mg to achieve deeper sedation could be administered. In case an additional period of deeper sedation was required, additional boluses of etomidate could be administered, up to a maximum dosage of 20 mg. The medication schedules for both general anesthesia and conscious sedation are schematically illustrated in Fig. 1.

### Ablation system

The ablation procedures were performed using a PFA system that includes a circular, over-the-wire ablation catheter equipped with nine electrodes. This PFA system generates energy applications consisting of four biphasic, bipolar pulse trains lasting 100–200 ms at 1.5 kV. Typically, four antral and four ostial applications were applied for each pulmonary vein (PV), in accordance with the instructions for use of the system.

### Ablation procedure

Femoral access was achieved aided by ultrasound guidance at the operator’s discretion. A multipolar (four or ten electrode) catheter was subsequently placed in the coronary sinus. Left atrial access was achieved using a fluoroscopically guided transseptal puncture, after which heparin was administered. Each PV was treated with at least eight applications (four distal antrum, four proximal antrum), while additional applications were allowed if isolation was not achieved after these applications. Isolation of the pulmonary veins was confirmed by testing for entrance and exit block using the ablation catheter. After confirming isolation of all PV’s, the catheters and sheaths were removed and hemostasis was achieved using manual pressure, a figure of eight suture and/or a vascular closure device. Protamine could be administered at the discretion of the operator. Patients were typically discharged from the hospital the same day or the day after the procedure.

### Follow-up

All patients were scheduled for a post ablation follow-up visit (online or at the outpatient clinic) at 3 months after the procedure. During this visit, patients were enquired for any adverse events.

### Endpoints

The primary efficacy endpoint of the study was defined as acute isolation of all PV’s during the procedure as demonstrated by entrance and/or exit block using the ablation catheter.

Secondary endpoints of the study included the following:

1. The need for conversion to GA in patients treated under CS.
2. Procedural characteristics, including total number of PFA-applications, total procedure time (vascular access to removal of all sheaths), and fluoroscopy time
3. Procedural complications including vagal reactions and coronary spasms
4. Short term follow-up efficacy and safety outcomes, including hospitalization duration and major and minor safety outcomes (within 30 days post-procedure). Major adverse events included death, cardiac tamponade, stroke, systemic thromboembolism, persistent phrenic nerve injury, and vascular access site complications requiring surgical intervention or transfusion.

### Data analysis and statistics

Data analysis was conducted at the St. Antonius Hospital using R, version 4.4.0 (R Foundation, Vienna, Austria). The statistical methods used were descriptive as this was an observational study which was not driven by a formal endpoint hypothesis. Baseline and procedure variables were presented as mean ± standard deviation (SD) or median [interquartile range (IQR)] for continuous variables, and frequencies with percentages for categorical variables. Comparisons between groups were performed using Student’s *t*-test for normally distributed continuous data, Mann–Whitney *U* test for non-normally distributed continuous data, and χ^2^ test for categorical data.

## Results

### Baseline characteristics

A total of 174 patients were treated with the circular, over-the-wire PFA catheter between January 29 and December 31, 2024, and included in this analysis. Most patients underwent PVI under general anesthesia (GA) (*n* = 132 (75.9%)), while the remainder (*n* = 43 (24.1%)) was treated under conscious sedation (CS). The cohort consisted of 67.2% males, with a mean age of 62.8 years and mostly paroxysmal AF (62.1%). There was a significant difference in the type of AF between the groups, with a higher percentage of paroxysmal AF in the CS group (76.2% vs. 57.6%, *p =* 0.047). The CHA_2_DS_2_ -VA score was 0 in 66 patients (37.9%), 1 in 46 patients (26.4%), and ≥ 2 in 61 patients (35.1%) and was not different between the two treatment strategies (*p =* 0.205). Other baseline characteristics are presented in Table 1 and were not different between the two treatment strategies.

**Table 1:** Baseline characteristics

	Conscious sedation (*n* = 42)	General anesthesia (*n* = 132)	*p*-value
Age, years	60.6 ± 9.6	63.4 ± 9.1	0.102
BMI, kg/m2	26.6 ± 3.1	27.4 ± 3.9	0.204
Female sex	12 (28.6)	45 (34.1)	0.635
Hypertension	14 (33.3)	49 (37.1)	0.794
Diabetes Mellitus	2 (4.8)	13 (9.9)	0.472
Obstructive sleep apnea	4 (9.5)	25 (18.9)	0.235
Chronic obstructive pulmonary disease	1 (2.4)	4 (3.0)	1.000
LVEF > 50%	34 (82.9)	99 (76.7)	0.536
Enlargement left atrium (LAVI > 35 ml/m^2^)	14 (40.0)	43 (35.5)	0.777
*AF baseline characteristics*			
Time since AF diagnosis, years	1.5 [0.6, 3.6]	1.9 [0.6, 4.7]	0.409
Type of AF			0.047
Paroxysmal AF	32 (76.2)	76 (57.6)	
Persistent AF	10 (23.8)	56 (42.4)	
CHA_2_DS_2_-VA score			0.205
0	20 (47.6)	46 (35.1)	
1	10 (23.8)	36 (27.5)	
2 +	12 (28.6)	49 (37.4)	
*Medication use prior to PVI*			
Class Ia antiarrhythmic drugs	11 (26.2)	26 (19.7)	0.497
Beta blocker	15 (35.7)	45 (34.1)	0.995
Amiodaron	8 (19.0)	14 (10.6)	0.243
Sotalol	12 (28.6)	49 (37.1)	0.409
Anticoagulant drugs	41 (97.6)	132 (100.0)	0.544

Data is presented as counts (percentages) for categorical data, means ± standard deviations for normally distributed continuous data and median [interquartile range] non-normally distributed continuous data. Abbreviations: *AF* atrial fibrillation, *LAVI* left atrial volume index, *LVEF* left ventricular ejection fraction, *PVI* pulmonary vein isolation

### Procedural characteristics and efficacy outcomes

The procedural characteristics are presented in Table 2. The total number of applications was 32 [IQR 32; 33] in the CS group vs. 33 [IQR 32; 36] in the GA group (*p =* 0.001). Additional applications, on top of the standard 32 applications, were performed in more than half of the total cohort: 47.6% of the CS group vs. 59.4% of the GA group (*p =* 0.248). These additional applications were most often performed in the LSPV in both groups (CS 39.0% vs. GA 43.3%, *p =* 0.764), followed by the LIPV in the GA group (CS 4.9% vs. GA 22.0% *p =* 0.024) and the RSPV in the CS group (CS 14.3% vs. GA 19.7%, *p =* 0.576). Procedure times were comparable between groups: CS group 42.0 [37.5; 46.0] min vs. GA group 39.0 [34.0; 46.0] min, *p =* 0.108. However, the LA dwell time was longer in the CS group 31.0 [28.0; 35.0] min vs. 28.0 [25.0; 32.8] min, *p =* 0.032. While the total fluoroscopy time was comparable between groups (CS 11.5 min vs. GA 12.0 min, *p =* 0.090), the dose area product (DAP) was higher in the GA group (7.0 [4.2; 11.8] Gy⋅cm2 vs. 4.5 [3.0; 8.0] Gy⋅cm2, *p =* 0.003).

**Table 2:** Procedural characteristics

	Conscious sedation (*n* = 42)	General anesthesia (*n* = 132)	*p*-value
Use of electroanatomic mapping system	0 (0.0)	2 (1.5)	1.000
Total number of applications	32.0 [32.0, 33.0]	33.0 [32.0, 36.0]	0.001
Electrocardioversion during procedure	12 (28.6)	44 (33.3)	0.700
Femoral closure			
Manual pressure	42 (100.0)	132 (100.0)	1.000
Figure of 8 suture	42 (100.0)	131 (99.2)	1.000
Closure device	0 (0.0)	4 (3.0)	0.582
Protamine use	0 (0.0)	60 (45.5)	< 0.001
Procedure time (min)	42.0 [37.5, 46.0]	39.0 [34.0, 46.0]	0.108
LA dwell time (min)	31.0 [28.0, 35.0]	28.0 [25.0, 32.8]	0.032
DAP (Gy⋅cm2)	4.5 [3.0, 8.0]	7.0 [4.2, 11.8]	0.003
Fluoroscopy duration (min)	11.5 [10.0, 12.2]	12.0 [10.0, 15.0]	0.090
Acute procedural success	42 (100.0)	132 (100.0)	1.000

Data is presented as counts (percentages) for categorical data, means ± standard deviations for normally distributed continuous data and median [interquartile range] non-normally distributed continuous data. *DAP* dose area product, *LA* left atrial

Acute isolation of pulmonary veins at the end of procedure was achieved in all patients in both groups.

Femoral closure was achieved with manual pressure in all patients, a figure of 8 suture in 99.2% of patients in the GA group vs. 100% in CS group (*p =* 1.000) and a closure device in 4 patients of the GA group (*p =* 0.582). Protamine was used in 60 (45.5%) of patients in the GA groups vs. none in the CS group (*P* < 0.001).

**Fig. 2:**
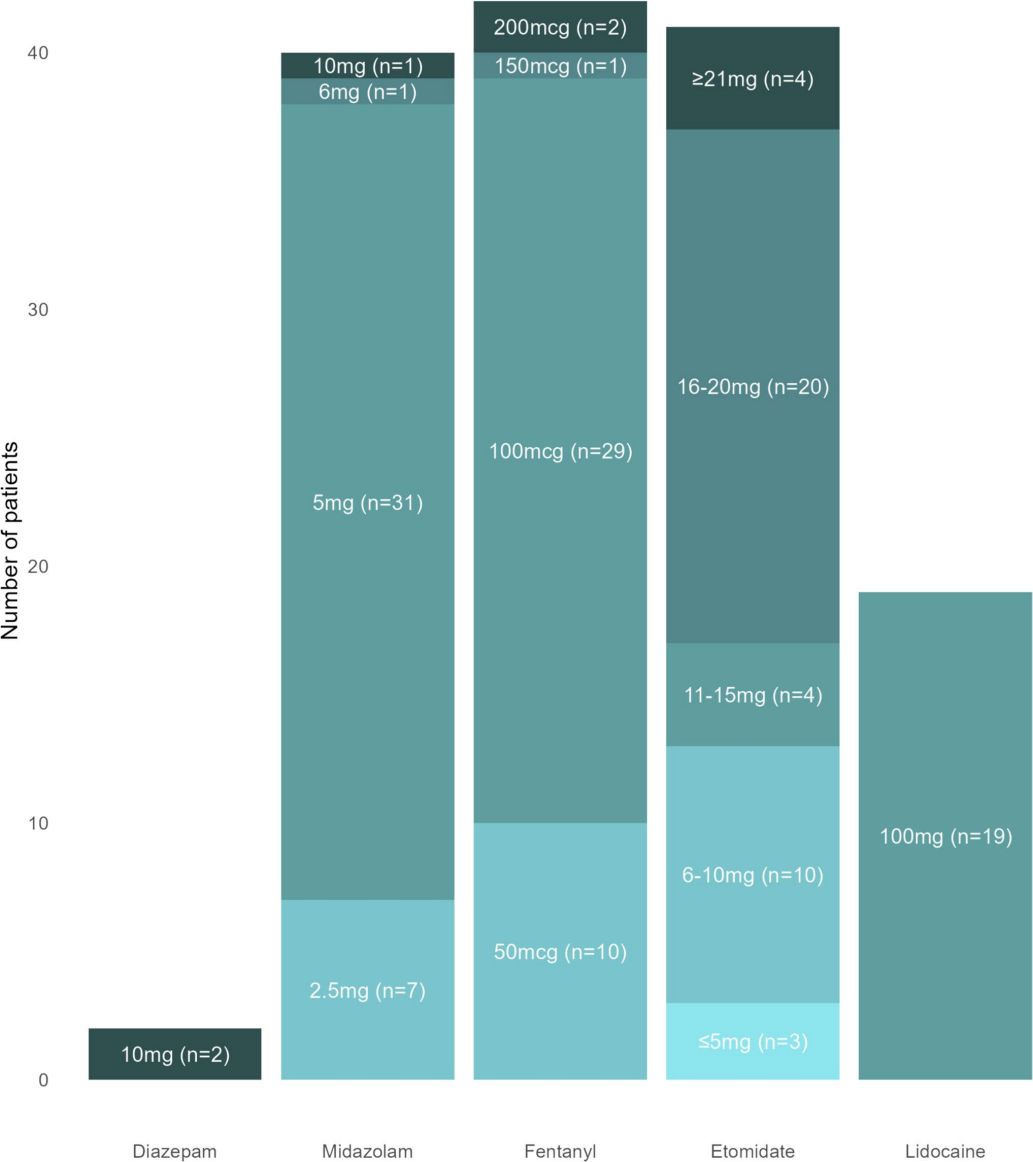
Dosage of sedatives and analgesics in 42 patients treated with the circular over-the-wire catheter under conscious sedation

Patients in the CS group received a median dose of 5 mg [5; 5] midazolam or 10 mg [10; 10] diazepam, 100 mcg [100; 100] fentanyl, and 20 mg [10; 20] etomidate during the procedure. No significant differences in dosing were observed between male and female patients. As of October 30, 2024, intravenous lidocaine was administered routinely to most patients in the CS group with a positive effect on coughing and discomfort in the majority of patients (18 out of 19). Figure 2 provides a detailed overview of the dosing strategies used across patients.

### Safety outcomes

None of the included patients in both groups experienced any intra-procedural complications. Additionally, no patients in the CS group developed symptoms that required conversion to GA. Of note, in one patient in the CS group, only four to five of the eight recommended standard applications per vein could be delivered due to significant discomfort, despite high doses of sedatives and opioids. Nonetheless, acute isolation of all PV’s was achieved after four to five applications per vein, and long-term follow-up (> 6 months) showed no recurrence of atrial arrhythmias. As this patient was treated before October 30, lidocaine was not administered during the procedure.

The majority of patients could be discharged from the hospital either the same day (42%) or the day after the procedure (58%) in both groups. One patient in the GA group had a prolonged hospital stay due to continuous oozing from the groin. This did not require surgical intervention or blood transfusion and did not lead to hemodynamic instability. The bleeding was eventually resolved with conventional therapy.

During a follow-up period of 30 days, no cases of death, tamponade, (transient or persistent) phrenic nerve injury, stroke, or thromboembolic complications were recorded. One patient in the GA group experienced a major bleeding complication with the need for re-hospitalization and blood transfusion. Ten patients (CS group 2 (4.8%) vs. GA group 8 (6.1%), *p =* N.S.) experienced a minor access site bleeding during primary hospital admission with the need for pressure bandage and prolonged bedrest. No other minor adverse events occurred during the 30-day follow-up (Table 3).

**Table 3:** Safety outcomes

	Conscious sedation (*n* = 42)	General anesthesia (*n* = 132)	*p*-value
Death	0 (0.0)	0 (0.0)	1.000
Tamponade	0 (0.0)	0 (0.0)	1.000
Thromboembolic events	0 (0.0)	0 (0.0)	1.000
Stroke	0 (0.0)	0 (0.0)	1.000
TIA	0 (0.0)	0 (0.0)	1.000
Phrenic nerve paralysis (persistent or transient)	0 (0.0)	0 (0.0)	1.000
Major bleeding	0 (0.0)	1 (0.8)	1.000
Minor bleeding	2 (4.8)	8 (6.1)	1.000
- Hb-loss > 1 mmol/L	0 (0.0)	3 (2.3)	0.760
- Prolonged hospitalization > 4 h	2 (4.8)	3 (2.3)	0.756
- Prolonged hospitalization < 4 h	0 (0.0)	2 (1.5)	1.000

Data is presented as counts (percentages) for categorical data. Abbreviations: *Hb* hemoglobin, *TIA* transient ischemic attack

## Discussion

Our study demonstrated an alternative workflow for performing PFA procedures using the circular, over-the-wire catheter under conscious sedation (CS), compared to traditionally used general anesthesia (GA) or continuous deep sedation [11, 12]. This workflow may be very relevant in geographies where anesthesiology is not readily available, which might hamper adoption of PFA.

In this study, procedural differences between the CS and GA group were small and of limited clinical significance. Acute isolation of the pulmonary veins was achieved in all patients of both sedation groups with only a higher median amount of total number of applications in the GA group (CS group 32 vs. GA group 33 applications). Moreover, although LA dwell times were a median of 3 min shorter in the GA group, the median total procedure times were comparable between the groups. This minor difference is presumably associated with the administration of sedatives between pulses by the treating electrophysiologist. There was no need to convert to GA in any patient treated under CS, and no differences in post-procedural safety were observed between the treatment groups.

CS is commonly used for catheter ablation in AF patients, particular during cryo- and RF-ablation procedures [13]. In this study, we report the outcomes of our CS approach in patients treated with PFA. This workflow involves the bolus-wise administration of sedatives and analgesics by the electrophysiologist, tailored to the continuously monitored level of patient comfort. Sedation levels are often defined according to the statement of The American Society of Anesthesiologists, which classifies sedation into four groups: minimal sedation, conscious sedation, deep sedation, and general anesthesia [14]. Instead of providing a continuous intravenous infusion of sedatives in the CS group to maintain a consistent state of sedation throughout the procedure, patients are managed on a sedation spectrum ranging from CS to short episodes of deep sedation during the ablation procedure with no need for specialized anesthesia personnel to continuously monitor for a secure airway, and without the need for intervention. This approach offers a patient-tailored solution while keeping the total dosage of sedation as low as possible. In our cohort, this method resulted in successful isolation of ablation targets in all patients, without the need for airway interventions or any procedure-related adverse events. This finding underscores the potential of performing PFA procedures with the circular, over-the-wire catheter without GA or continuous deep sedation, thereby reducing the risk of anesthesia-related complications in patients without pre-existing severe pulmonary disorders. Furthermore, our approach could serve as a model for other centers facing challenges in implementing PFA-procedures due to limited anesthetic support.

During procedures with PFA, diaphragmatic contraction by phrenic nerve stimulation and dry cough might influence catheter stability, patient comfort, and clinical outcomes. Our results demonstrated 100% acute pulmonary vein isolation in the CS group, suggesting no immediate concerns regarding catheter stability. However, long-term outcome data are not yet available. While outcomes like patient comfort, degree of diaphragmatic contractions, and coughing were not quantified, most patients experienced those symptoms at least to some degree.

The introduction of intravenous lidocaine bolus infusion significantly reduced symptoms of coughing and discomfort in the majority of patients. Although the exact mechanism by which lidocaine suppresses coughing is not completely understood, it is commonly used by anesthesiologists to facilitate both intubation and extubation [15]. Studies have demonstrated a reduced incidence of coughing during these moments in surgical patients. Furthermore, a systematic review involving over 1500 surgical patients reported no adverse events directly attributable to lidocaine use, and no adverse effects were observed in our own cohort [9, 10]. Another possible strategy in reducing coughing during PFA procedures was posed in a small study in 28 patients treated with a circular PFA system (AccuPulse, AccuPulse Medical Technology, Suzhou, China) [16]. Patients were treated under CS using midazolam and fentanyl and the effect of respiratory control on diaphragmatic contractions was investigated. This study showed that a workflow solution with the application of PFA pulses at the end of the expiratory phase in the respiratory cycle could reduce the severity of diaphragmatic contraction and dry cough. These and other solutions might offer improvements to our proposed workflow, making the implementation of our workflow easier with possibly lower need for sedatives.

### Limitations

This study is limited by its observational, non-randomized design, which could have introduced selection bias. We believe, however, that this risk is minimized by the fact that patients were assigned to a specific sedation and ablation method by the local planning department without any influence of the treating physician, although patients with severe obstructive sleep apnea or significant pulmonary disease were excluded from treatment under CS. Another limitation is that the study did not include patient comfort as an outcome measure, due to the retrospective nature of this analysis. Future studies could prospectively collect data on patient comfort to assess for any differences between sedation methods. Nonetheless, based on experience from both the treating physicians and EP staff, the CS procedure was well tolerated in all but one patient and none of the treated patients showed any signs of discomfort after the procedure. Lastly, this study does not provide evidence on the effects of sedation strategy on long-term efficacy outcomes, as data collection is still ongoing. Previous studies on the relationship between choice of anesthesia and arrhythmia-free survival in pulmonary vein isolation with thermal ablation techniques have shown conflicting results [17–19]. This indicates that further data are required to clarify the impact of anesthesia type on ablation outcomes.

## Conclusion

In this article, we showed a feasible new workflow for performing PFA procedures using CS in patients with atrial fibrillation. In our cohort, this workflow showed that the procedural efficacy is comparable to general anesthesia, without compromising on safety. Further research is needed to support our findings in a randomized trial and investigate long-term and patient-related outcomes like procedural comfort, coughing, and patients’ preferences.

## Abbreviations

AF: Atrial fibrillation
CS: Conscious sedation
GA: General anesthesia
LAVI: Left atrial volume index
PFA: Pulsed field ablation
PV: Pulmonary vein
PVI: Pulmonary vein isolation
